# Risk Profiles and Treatment Patterns in Atrial Fibrillation Patients with Chronic Kidney Disease Receiving or not Receiving Anticoagulation Therapy

**DOI:** 10.1055/s-0044-1780529

**Published:** 2024-02-23

**Authors:** Reinhold Kreutz, Gilbert Deray, Jürgen Floege, Marianne Gwechenberger, Kai Hahn, Andreas R. Luft, Pontus Persson, Christoph Axthelm, Juerg Hans Beer, Jutta Bergler-Klein, Nicolas Lellouche, Jens Taggeselle, Jan Beyer-Westendorf

**Affiliations:** 1 Institute of Clinical Pharmacology and Toxicology, Charité – Universitätsmedizin Berlin, Berlin, Germany; 2 Department of Nephrology, Pitié-Salpêtrière Hospital, Paris 6 University, Paris, France; 3 Division of Nephrology and Clinical Immunology, RWTH Aachen University Hospital, Aachen, Germany; 4 Division of Cardiology, University Department of Internal Medicine II, Medical University of Vienna, Vienna, Austria; 5 Nephrologische Praxis, Dortmund, Germany; 6 Center for Neurology and Rehabilitation, Klinik für Neurologie, Universitätsspital Zürich, Switzerland and Cereneo, Vitznau, Switzerland; 7 Institut für Vegetative Physiologie, Charité – Universitätsmedizin Berlin, Berlin, Germany; 8 Cardiologicum Pirna and Dresden, Dresden, Germany; 9 Department Innere Medizin, Baden Switzerland and Center of Molecular Cardiology, Kantonsspital Baden, University of Zürich, Zürich, Switzerland; 10 Service de Cardiologie 1, Centre Hospitalier Universitaire Henri Mondor, Créteil, France; 11 Praxis Dr. med. Jens Taggeselle, Markkleeberg, Germany; 12 Thrombosis Research Unit, Division Haematology, Department of Medicine I, University Hospital Carl Gustav Carus Dresden, Dresden, Germany

**Keywords:** atrial fibrillation, chronic kidney disease, anticoagulation, elderly, direct oral anticoagulants

## Abstract

**Background** Patients with atrial fibrillation (AF) and chronic kidney disease (CKD) are at high risk for both thromboembolism and bleeding events. The latter induces a potential reason for withholding oral anticoagulation (OAC) despite an indication for prophylaxis of thromboembolic events.

**Methods** AF patients with CKD (estimated glomerular filtration [eGFR] rate between 15 and 49 mL/min per 1.73 m ^2^ ) were included in a prospective international registry in Europe between 2016 and 2020, that is, XARENO (factor XA inhibition in renal patients with nonvalvular atrial fibrillation observational registry). The study enrolled adult patients treated at the discretion of physicians with rivaroxaban, vitamin K antagonists (VKA), or without OAC (w/oOAC). Here, we report a prespecified explorative baseline comparison between patients receiving OAC or no OAC within XARENO.

**Results** In total, 1,544 patients (mean age: 78.2 years, mean eGFR: 36.2 mL/min) were studied (rivaroxaban *n* = 764, VKA *n* = 691, w/oOAC *n* = 89). Patients in the w/oOAC group were older and had a similar stroke (mean CHA _2_ DS _2_ -VASc score 4.0) but higher bleeding risk (mean modified Hypertension, Abnormal Renal/Liver Function, Stroke, Bleeding History or Predisposition, Labile INR, Elderly, Drugs/Alcohol Concomitantly score 2.5 vs. 1.8) compared with the OAC groups. The distribution of comorbidities including hypertension, diabetes, and heart failure was similar. Treatment with antiplatelet drugs was fivefold more frequent in the w/oOAC group.

**Conclusion** Only 5.8% of the overall population of AF patients with advanced CKD received no OAC. These patients were older and had a higher bleeding risk, which might explain this decision, but which contrasts with the more frequent use of antiplatelet drugs in this vulnerable group of patients.

## Introduction

Patients with atrial fibrillation (AF) and chronic kidney disease (CKD) are at high risk for both thromboembolism and bleeding events. 1 2 The latter is a potential reason for withholding oral anticoagulation (OAC) despite an indication for prophylaxis of ischemic stroke and systemic embolism in these patients. 1 2 In the pivotal randomized controlled trials (RCTs) that compared direct oral anticoagulants (DOACs) and vitamin K antagonist (VKA) for the prevention of thromboembolism in nonvalvular AF, patients with an estimated creatinine clearance (CrCl) <25 to 30 mL/min were excluded and only a small proportion of patients in these RCTs (overall approximately 19%) had moderate kidney function impairment (CrCl < 50 mL/min). 2 3 4 This applied also to the ROCKET-AF (Rivaroxaban Once Daily Oral Direct Factor Xa Inhibition Compared with Vitamin K Antagonism for Prevention of Stroke and Embolism Trial in Atrial Fibrillation) RCT that compared treatment with 20 mg once daily rivaroxaban with warfarin for the prevention of stroke and systemic embolism in 14,264 patients with nonvalvular AF. 5 In this trial, a reduced daily dose of 15 mg rivaroxaban was used in 2,950 patients (20.7% of the study population) with moderate kidney function impairment, that is, CrCl between 30 and 49 mL/min. 6 A subgroup analysis in this group indicated that these patients were older (mean age 79 years) as compared with the rest of the study population (mean age 73 years) and had higher event rates irrespective of study treatment. There was no heterogeneity in treatment effect across dosing groups in ROCKET-AF as treatment with 15 mg once daily rivaroxaban induced similar results and was noninferior to warfarin treatment. 6 However, patients with severe CKD, that is, CrCl <30 mL/min were excluded from ROCKET-AF. 5 Thus, the use of anticoagulant treatment according to the CHA _2_ DS _2_ -VASc (congestive heart failure, hypertension, age ≥ 75 years, diabetes mellitus, stroke or transient ischemic attack [TIA], vascular disease, age 65 to 74 years, and sex category) score 4 remains controversial for patients with CKD categories 4 and 5. Conversely, several observational studies support OAC with VKA in patients with advanced CKD and AF 7 8 and comparative observational studies suggest that the benefits of DOAC over VKA treatment may also extend to patients with eGFR values < 30 mL/min/1.73 m ^2^ . 9 10 In addition to stroke prevention, decline versus preservation of kidney function has recently gained interest. Several studies have suggested a potential benefit of DOACs versus VKA by improving kidney outcomes including a slower decline of kidney function over time and the reduced development of CKD stage 5. 11 12 However, the hypothesis of VKA-related acceleration of kidney impairment is still a matter of debate and supporting data are scarce. We conducted a prospective real-world registry in patients with AF and advanced CKD to compare the effectiveness of rivaroxaban versus VKA treatment for kidney outcomes (progression of CKD) and net-clinical benefits (stroke and other thromboembolic events, major bleeding, and all-cause mortality). 13 In this XARENO (XA inhibition in renal patients with non-valvular atrial fibrillation observational registry) registry, AF patients with a baseline eGFR between 15 and 49 mL/min per 1.73 m ^2^ were included, if they were treated with either rivaroxaban, VKA, or no anticoagulation. 13 Here, we compare patient characteristics and differences in risk profiles between the cohorts of patients receiving OAC or not receiving OAC in the XARENO study to evaluate treatment selection patterns in this vulnerable AF population.

## Methods

The design of the XARENO study has been recently reported. 13 XARENO was conducted as an international multicentre, prospective, and noninterventional registry in Europe including patients from Germany, Austria, Switzerland, France, Belgium, and Luxembourg. Management of patients was at the discretion of the participating physicians. The study is registered with clinical trials.gov (NCT02663076).

Inclusion criteria were a diagnosis of nonvalvular atrial fibrillation as diagnosed by the participating physicians, adult age (≥18 years), and a baseline eGFR between 15 and 49 mL/min per 1.73 m ^2^ as estimated by the chronic kidney disease epidemiology collaboration equation. 14 Patients treated either with rivaroxaban or VKA or no OAC at the discretion of attending physicians were eligible for inclusion. Patients should have been treated with either the recommended 15 mg once daily dose of rivaroxaban or VKA for at least 3 months before enrolment. A third group of patients without OAC (w/oOAC) were also eligible and enrolled for explorative analysis to obtain a full picture of anticoagulation patterns because we anticipated that physicians could be inclined to withhold OAC in selected patients with more advanced CKD or a higher comorbidity burden.

Prespecified follow-up is at least 12 months followed by a planned extended data collection period for up to 2 additional years. Effectiveness outcomes include progression of CKD and other efficacy and safety outcomes including a combined net-clinical benefit outcome (stroke and other thromboembolic events, myocardial infarction or acute coronary syndrome), major bleeding (according to the International Society of Thrombosis and Haemostasis (ISTH] classification 15 ), and all-cause mortality and its individual components. All outcomes will be analyzed in intention-to-treat analysis (ITT) under the initial treatment (safety population). However, according to protocol, 13 the final primary outcome statistical analysis will focus on the comparison between rivaroxaban and VKA groups and will be reported elsewhere. This analysis requires additional propensity score matching to account for potential allocation bias between the rivaroxaban and VKA groups. To allow for meaningful comparisons between rivaroxaban and VKA, planned prospective treatment with apixaban, dabigatran, edoxaban or any nonapproved experimental anticoagulant drug at baseline excluded patients from participation. Additional exclusion criteria included chronic treatment with parenteral anticoagulants and current or expected kidney-replacement therapy within the next 3 months.

The analysis reported here relates to comparisons between rivaroxaban and VKA cohorts and, as an explorative analysis, to comparisons between pooled rivaroxaban/VKA patients versus patients not receiving anticoagulation at baseline (w/oOAC patients).

For the simultaneous evaluation of ischemic stroke, mortality, and bleeding risk, we used the GARFIELD-AF (Global Anticoagulant Registry in the FIELD–Atrial Fibrillation) risk tool. 16 Additionally, separate risk estimations were performed for stroke by the CHA _2_ DS _2_ -VASc score, 4 and bleeding risk by the modified HAS-BLED score (hypertension, abnormal kidney/liver function, stroke, bleeding history or predisposition, labile international normalized ratio, elderly [>65 years], and drugs/alcohol concomitantly). 15 17 18

Statistical analyses were performed by using the software package SAS release 9.4 or higher (SAS Institute Inc., Cary, NC, United States) and were of descriptive and exploratory nature. The analysis focused on the difference between the w/oOAC and the two groups with OAC, that is, the rivaroxaban and VKA groups combined. Statistical significance was considered with *p* -values <0.05.

## Results

**Fig. 1:**
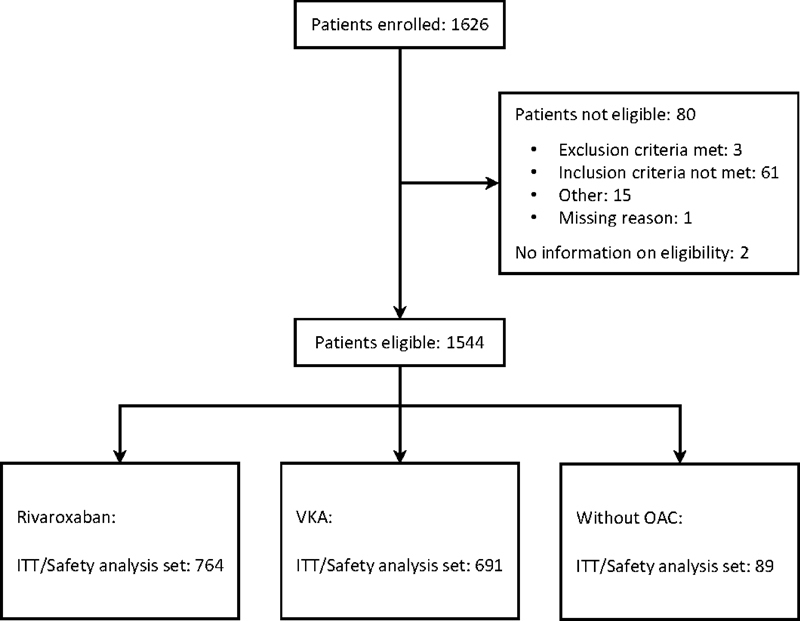
Flow diagram with patient disposition in the XARENO registry

Of the 1,626 patients initially enrolled into XARENO across 117 sites between April 2016 and January 2020, 1544 were eligible to be included in the observation phase ( Fig. 1 ). The main reason for noneligibility was that an inclusion criterion was not met, which occurred in 61 patients. Of the remaining 1,544 eligible patients, 764 were treated with rivaroxaban, 691 with VKA, and 89 were enrolled into the w/oOAC cohort.

The baseline characteristics of patients in the overall study population and in the separate groups are shown in Table 1 . The overall mean age (± standard deviation [SD]) was 78.2 ± 7.6 years. Patients in the w/oOAC group were significantly older with a mean age of 80.6 ± 9.2 years as compared with both groups with OAC combined ( *p* = 0.01). The proportion of women overall was 44.2% and similar in groups. The mean eGFR in the study population was 36.2 ± 10.0 mL/min/1.73 m ^2^ . The mean eGFR was significantly lower in the w/oOAC group, but this was largely because the rivaroxaban group had higher eGFR levels compared with VKA recipients, which drove the statistical difference between patients with or without anticoagulation ( Table 1 ). Accordingly, the highest proportion of patients in CKD stage 4, that is, with eGFR values below 30 mL/min/1.73 m ^2^ , was observed in the w/oOAC group (37.1%) and the lowest in the rivaroxaban group (13%).

**Table 1:** Baseline characteristics of patients in the intention-to-treat analysis

	Total ( *n* = 1,544)	Rivaroxaban ( *n* = 764)	Vitamin K antagonist ( *n* = 691)	Without anticoagulation ( *n* = 89)	*p* -Value
Age	78.2 ± 7.6	77.7 ± 7.4	78.5 ± 7.6	80.6 ± 9.2	**<0.01**
≥75 y old (%)	1,123 (72.7)	540 (70.7)	516 (74.7)	67 (75.3)	0.26
Women (%)	682 (44.2)	349 (45.7)	294 (42.5)	39 (43.8)	0.95
eGFR mL/min per 1.73 m ^2^	36.2 ± 10.0	39.1 ± 8.5	33.2 ± 10.6	33.6 ± 10.1	**0.02**
CKD stage ≥ 3b = eGFR <45 mL/min per 1.73 m ^2^ (%)	1,084 (70.2)	501 (65.6)	515 (74.5)	68 (76.4)	**0.46**
CKD stage ≥ 4 = eGFR <30 mL/min per 1.73 m ^2^ (%)	380 (24.6)	99 (13.0)	248 (35.9)	33 (37.1)	**<0.01**
BMI (kg/m ^2^ )	29.0 ± 5.6	29.1 ± 5.7	29.0 ± 5.5	27.9 ± 5.8	0.07
Hypertension (%)	1239 (80.2)	609 (79.7)	557 (80.6)	73 (82.0)	0.60
Diabetes (%)	619 (40.1)	300 (39.3)	288 (41.7)	31 (34.8)	0.28
Myocardial infarction (%)	204 (13.2)	91 (11.9)	99 (14.3)	14 (15.7)	0.47
Heart failure (%)	338 (21.9)	166 (21.7)	157 (22.7)	15 (16.9)	0.23
Ischemic stroke (%)	119 (7.7)	63 (8.2)	49 (7.1)	7 (7.9)	0.96
Hemorrhagic stroke	9 (0.6)	3 (0.4)	3 (0.4)	3 (3.4)	**<0.01**
TIA	51 (3.3)	22 (2.9)	26 (3.8)	3 (3.4)	0.97
Systemic embolism	2 (0.1)	0	2 (0.3)	0	0.72

Abbreviations: BMI, body mass index; CKD, chronic kidney disease; eGFR, estimated glomerular filtration rate; TIA, transient ischemic attack; VKA, vitamin K antagonist.

Significant *p* -values < 0.05 are shown in bold.

Note: *p* -Value for the comparison between the group without oral anticoagulation and the rivaroxaban and VKA groups combined.

Note: Data are presented as mean ± standard deviation or percentages, as appropriate.

**Fig. 2:**
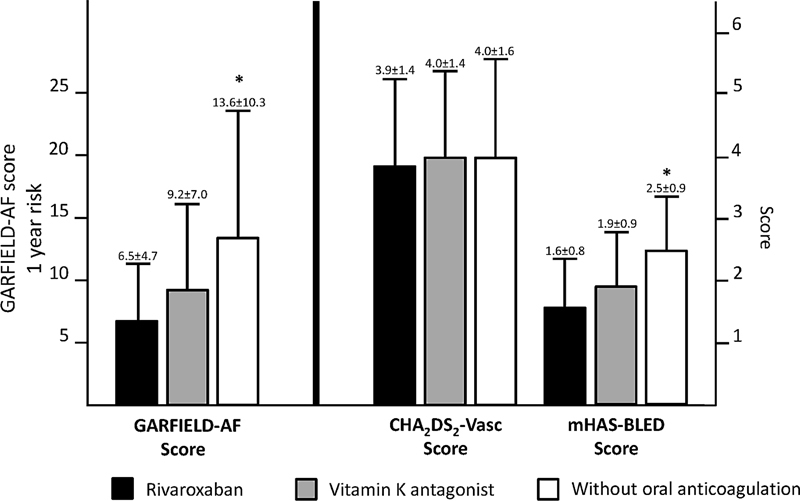
Estimated risk according to treatment groups in the XARENO registry. Note: * *p* < 0.01 for the comparison between the group without oral anticoagulation and the rivaroxaban and VKA groups combined. Data are presented as mean ± standard deviation.

The distribution of BMI and comorbidities including hypertension, diabetes, myocardial infarction, heart failure, and a history of stroke, TIA or systemic embolism was similar between groups with the exception that the rate of patients with a history of a hemorrhagic stroke (overall rate being low) was higher in the w/oOAC group. Hypertension (80.2%) and diabetes (40.1%) were the most frequent comorbidities. The GARFIELD-AF risk score analysis revealed the highest scores in the w/oOAC group indicating a significantly higher estimated combined 1-year risk for stroke, bleeding, and mortality in this group ( Fig. 2 ). Mean HAS-BLED score values were also significantly higher in the w/oOAC group, but mean CHA _2_ DS _2_ -VASc score values were comparable between groups ( Fig. 2 ). However, the proportion of patients with very high stroke risk, that is, CHA _2_ DS _2_ -VASc score >5, was significantly higher in the w/oOAC group (21.3%) compared with the OAC groups (11.1% in the rivaroxaban and 12.9% in the VKA group, *p* < 0.01).

The patterns of cardiovascular pharmacotherapy are summarized in Table 2 . The use of angiotensin-converting enzyme inhibitors, angiotensin receptor blockers, and statins was similar between groups. The use of β-blockers and verapamil was significantly lower in the w/oOAC, with β-blockers (78.3% in OAC vs. 66.3% in w/oOAC) being the most widely used antiarrhythmic drug, whereas amiodarone (21.1 vs. 19.1%), digitalis (11.3 vs. 4.5%), and verapamil (1.0 vs. 0%) were less commonly used ( Table 2 ).

**Table 2:** Cardiovascular pharmacotherapy of patients at baseline in the intention-to-treat analysis

	Total ( *n* = 1,544)	Rivaroxaban ( *n* = 764)	Vitamin K antagonist ( *n* = 691)	Without anticoagulation ( *n* = 89)	*p* -Value
ACE-inhibitors	539 (34.9)	280 (36.6)	227 (32.9)	32 (36.0)	0.84
ARB	553 (35.8)	269 (35.2)	248 (35.9)	36 (40.4)	0.36
β-blockers	1208 (78.2)	598 (78.3)	551 (79.7)	59 (66.3)	**<0.01**
CCB	461 (29.9)	238 (31.2)	201 (29.1)	22 (24.7)	0.27
Verapamil	15 (1.0%)	8 (1.0)	7 (1.0)	0	**<0.01**
Diuretics	1239 (80.2)	584 (76.4)	590 (85.4)	65 (73.0)	0.07
Digoxin or digitoxin	162 (10.5)	86 (11.3)	72 (10.4)	4 (4.5)	0.06
Amiodarone	314 (20.3)	161 (21.1)	136 (19.7)	17 (19.1)	0.76
Statins	854 (55.3)	416 (54.5)	393 (56.9)	45 (50.6)	0.34
Any antiplatelet drug	266 (17.2)	103 (13.5)	100 (14.5)	63 (70.8)	**<0.01**
Low-dose aspirin	196 (12.7)	60 (7.9)	79 (11.4)	57 (64.0)	**<0.01**
Clopidogrel	113 (7.3)	61 (8.0)	42 (6.1)	10 (11.2)	0.16
Dual antiplatelet therapy	45 (2.9)	18 (2.4)	23 (3.3)	4 (4.5)	0.36

Abbreviations: ACE, angiotensin-converting enzyme; ARB, angiotensin receptor blocker; CCB, calcium channel blocker.

Note: *p* -Value for the comparison between the group without oral anticoagulation and the rivaroxaban and VKA groups combined. Significant *p* -values < 0.05 are shown in bold. Number of patients and percentage (in brackets) are shown.

Overall, 17.2% of the study population was treated with antiplatelet drugs and most frequently with low-dose aspirin (12.7%). The use of antiplatelet drugs was approximately fivefold higher (70.8%) in the w/oOAC group as compared with the two other OAC groups. Still, 13.5% in the rivaroxaban and 14.5% in the VKA cohort were using antiplatelet drugs concomitantly despite receiving therapeutic anticoagulation.

## Discussion

The group of very old patients represents the fastest-growing population in many regions in the world including European countries. 19 In such patients, AF is the most common chronic cardiac arrhythmia 4 with a prevalence of at least 30% in the population older than 80 years. 19 20 CKD is also increasingly prevalent in the general aging population and shares common risk factors with AF. Older age, hypertension, and diabetes are important risk factors to develop either AF or CKD. 1 21 These were also the most prevalent comorbidities in the XARENO study.

Over the last decade, large AF registries were set up, largely driven by the widespread use of DOAC. However, these registries included only a small proportion of patients with concomitant CKD stages 4 and 5. In the large GARFIELD-AF registry, physicians classified 10.9% of patients as having moderate-to-severe CKD 21 ; these patients had a mean age of 78 years, similar to the current study. However, only a small portion (1.7%) of the overall GARFIELD-AF population (564 from 33,024 patients) were diagnosed with advanced CKD 4 or 5. The PREFER (Prevention of Thromboembolic Events–European Registry in Atrial Fibrillation) multicentre registry of AF patients also included only a small portion of patients with chronic kidney failure, while CKD stages were not further classified. 22

XARENO was, therefore, designed as the first prospective registry specifically dedicated to enrolling AF patients with advanced stages of CKD. In this often multimorbid and frail population, concerns remain about the benefit of stroke prevention when balanced against the risk of major bleeding. 1 2 AF patients with CKD, particularly older patients, are susceptible to a higher risk of bleeding. Not surprisingly, both kidney function impairment and older age are components of the HAS-BLED score to estimate the risk of major bleeding. 23 The same applies to the use of antiplatelet drugs. It is, therefore, surprising that, in XARENO, more than 70% of patients in the w/oOAC cohort were treated with antiplatelet drugs, which is approximately fivefold higher compared with the OAC cohorts in XARENO.

The increased bleeding risk in CKD has at least in part been attributed to impaired platelet function. 2 It follows that patients deemed ineligible to receive OAC would also be ineligible to receive antiplatelet drugs. A hesitance to use antiplatelet drugs instead of OAC would also be in agreement with the higher calculated GARFIELD-AF and HAS-BLED scores in our w/oOAC cohort, also because the HAS-BLED score performs well in predicting bleeding risk in AF patients who are treated with antiplatelet therapy alone. 23 Still, the high proportion of patients receiving antiplatelet drugs in all XARENO cohorts but especially the w/oOAC subset contradict this expectation.

A possible explanation might be that the baseline risk of these frail patients is already high without any antithrombotic treatment so that additional risk increases are seen of less importance compared with the antithrombotic benefits. Such a hypothesis would be supported by a recent real-world study in hospitalized patients showing even in very old (mean age 85.8 years) frail patients with AF and a high burden of comorbidities that the risk of bleeding between patients receiving or not OAC did not significantly differ. 20 Additionally, the bleeding risk was also not different for patients with eGFR values above or below 50 mL/min. The PREFER registry of AF patients also indicated a similar risk of major bleeding in very old AF patients on OAC and in those on antiplatelet therapy or without antithrombotic treatment. 22

A recent RCT in 985 older Japanese patients with AF 24 (mean age 86.6 years) included also patients with a CrCl as low as ≥15 mL/min resulting in a mean CrCl of 36.3 mL/min. Patients were randomized 1:1 to either once daily low-dose edoxaban (15 mg) or placebo. OAC in this trial resulted in a significant risk reduction (hazard ratio: 0.34, 95% confidence interval [CI]: 0.19–0.61; *p* < 0.001) in the rate of stroke and systemic embolism, while the risk for major bleeding was numerically but not statistically significantly higher (hazard ratio: 1.87, 95% CI: 0.90–3.89; *p* = 0.09) in the OAC group and death rates were similar between groups. 24 However, 53% of patients in the edoxaban group and 55% of patients in the placebo group were users of antiplatelet drugs. Thus, about every second patient in the edoxaban group was treated with combined OAC and antiplatelet treatment.

Taken together, our data confirm that prescribers seem to assign antiplatelet drugs a potential for thromboembolic protection in AF patients with CKD who are not seen as candidates for anticoagulation. However, antiplatelet drugs also carry a risk for bleeding and our study clearly shows that such patients are at higher bleeding risk at baseline already. Further studies are needed to evaluate whether antiplatelet therapy provides an acceptable risk-benefit ratio compared with anticoagulation in AF patients with advanced CKD stages.

The presented report is a prespecified descriptive analysis of treatment patterns in XARENO. 13 According to the noninterventional character of this real-world study, the choice of therapy (rivaroxaban or VKA or w/oOAC) was at the discretion of the participating physicians, which is a limitation of our analysis. Overall, only in a relatively small (5.8%) fraction of the overall study population, attending physicians withheld OAC treatment in AF patients with advanced CKD. In the w/oOAC group of patients, physicians were also less frequently using drugs related to heart rate control, that is, β-blockers, verapamil, and digitalis medications. Thus, in contrast to the frequent use of antiplatelet drugs, this may reflect a more cautious selection of some other cardiovascular drugs with anticipated side effects, for example, bradycardia, in this group of patients. This notion is supported against the background that the mean heart rate at baseline was similar in all groups (74.0, 74.1, and 75.3 beats per minute in the rivaroxaban, VKA, and w/oOAC group, respectively).

Because of a potential selection bias during enrolment into the study, we do not know whether the observed frequency of patients w/oOAC reflects broader clinical practice in the participating countries. The comparison of patient characteristics and risk profiles across all three groups reveals that patients in the rivaroxaban group were younger, had better kidney function, and lower risk profiles at baseline compared with w/oOAC patients but also, although less pronounced, in comparison to VKA patients. Therefore, the planned outcome analysis comparing the rivaroxaban and VKA groups will need to account for group differences between rivaroxaban and VKA groups by propensity score matching and to account for potential allocation bias between the rivaroxaban and VKA groups. 13

## Conclusion

In conclusion, XARENO provides a current view on selection patterns for or against OAC in AF patients with advanced stages of CKD in Western Europe. The data suggest that anticoagulation is rarely withheld in this frail population and that usage of antiplatelet drugs is frequent in those not prescribed OAC. Finally, the use of antiplatelet drugs was also observed in approximately 14% of patients in the OAC cohorts, raising concerns about the need for and the safety of combining OAC and antiplatelet drugs in this setting. In the absence of compelling indications for the use of antiplatelet therapy such as in patients with acute coronary symptoms, aiming for OAC monotherapy 4 25 in older AF patients with advanced CKD appears reasonable.
